# Cytokinesis-Block Micronucleus Cytome Assay Evolution into a More Comprehensive Method to Measure Chromosomal Instability

**DOI:** 10.3390/genes11101203

**Published:** 2020-10-15

**Authors:** Michael Fenech

**Affiliations:** 1 Genome Health Foundation, North Brighton, SA 5048, Australia; michael.fenech@unisa.edu.au; 2 Centre for Healthy Aging and Wellness, Faculty of Health Science, Universiti Kebangsaan Malaysia, Jalan Raja Muda Abdul Aziz, Kuala Lumpur 50300, Malaysia; 3 University of South Australia, School of Pharmacy and Medical Sciences, Adelaide, SA 5000, Australia

**Keywords:** micronucleus, nucleoplasmic bridge, nuclear bud, cytokinesis-block, cytome, chromosomal instability, cGAS-STING

## Abstract

This review describes the cytokinesis-block micronucleus (CBMN) cytome assay and its evolution into a molecular cytogenetic method of chromosomal instability (CIN). Micronuclei (MNi) originate from whole chromosomes or chromosome fragments that fail to segregate to the poles of the cell during mitosis. These lagging chromosomes are excluded from the daughter nuclei and are enveloped in their own membrane to form MNi. The CBMN assay was developed to allow MNi to be scored exclusively in once-divided binucleated cells, which enables accurate measurement of chromosome breakage or loss without confounding by non-dividing cells that cannot express MNi. The CBMN assay can be applied to cell lines in vitro and cells such as lymphocytes that can be stimulated to divide ex vivo. In the CBMN assay, other CIN biomarkers such as nucleoplasmic bridges (NPBs) and nuclear buds (NBUDs) are also measured. Use of centromere, telomere, and chromosome painting probes provides further insights into the mechanisms through which MNi, NPBs and NBUDs originate. Measurement of MNi is also important because entrapment within a micronucleus may cause chromosomes to shatter and, after nuclear reintegration, become rearranged. Additionally, leakage of DNA from MNi can stimulate inflammation via the cyclic GMP-AMP Synthase—Stimulator of Interferon Genes (cGAS-STING) DNA sensing mechanism of the innate immune system.

## 1. Background

Interest is growing in high-content assays that can reliably measure chromosomal instability (CIN) because of its important role in causing developmental defects, cancer, and accelerated aging [1,2,3,4]. Chromosomal aberrations can be measured directly using metaphase analysis of chromosomes or indirectly by interphase cytogenetic techniques that analyse the DNA content of nuclei and abnormalities of their morphology. One of the most prominent of these nuclear aberrations are micronuclei (MNi), which were discovered more than 100 years ago. This brief review explains the origin of MNi and the evolution of their measurement, together with other nuclear anomalies, into a multi-endpoint assay of CIN.

## 2. The Origin of Micronuclei

In 1902, Theodor Boveri (and David Hansemann before him) proposed the following hypothesis: “A malignant tumour cell is a cell with a specific abnormal chromosome constitution.” [5]. Boveri also suggested the hypothesis that abnormal chromosome numbers are due to mitotic errors:

“Nonetheless, I regard irregularities of mitosis as the usual way in which a nucleus of inappropriate composition is generated. I would like to say a few more words about this. So-called asymmetrical mitoses, which do not appear to be rare, may have much the same effect as multipolar mitoses. Asymmetrical mitoses, as the analysis of suitable material has shown, arise when the fibres associated with one of the two centrospheres do not form an attachment to all the chromosomes. The chromosomes that are attached to only one set of fibres—or, if division of the chromosome has already begun, the two unseparated daughter chromosomes—then pass to one of the daughter cells, whereas the other daughter cell remains devoid of these elements. It is precisely these aberrations that can easily lead to a marked numerical excess of one type of chromosome relative to the others, if that is what is required.”

[5]

Boveri’s hypotheses have been proven to be correct, especially with regards to the central role of chromosomal instability and aneuploidy in cancer. However, in those early years, there was no reported appreciation that chromosome mal-segregation during mitosis may also result in whole chromosomes or chromosome fragments being left outside the daughter nuclei and isolated from them by the formation of their own nuclear membrane (or “nuclear envelope”) to form MNi. MNi were first reported in red blood cells (RBCs) more than 100 years ago by haematologists Howell and Jolly and are also known as Howell–Jolly bodies [6]. They were shown to be increased in splenectomised subjects with megaloblastic anaemia, sickle cell anaemia, in those exposed to ionising radiation, and those with folate and/or vitamin B-12 deficiency [7,8,9].

MNi in RBCs originate in the dividing normoblast precursor cells in the bone marrow. The nucleus in the normoblast is ejected during the RBC maturation process but MNi are retained. The RBCs with or without MNi leave the bone marrow and enter the peripheral bloodstream. Measurement of MNi in RBCs in the bone marrow and peripheral blood has become one of the most widely used tests for measuring genotoxicity in rodents and humans [10,11]. Eventually, it became evident that MNi are also expressed in bone marrow precursors of leukocytes and peripheral blood lymphocytes [12].

The use of peripheral blood lymphocytes has the distinct advantage that they can be studied in vivo as well as ex vivo and in in vitro assays because of the availability of well-developed techniques to stimulate lymphocytes to divide with mitogens, which enables the expression of MNi caused by mitotic errors and structural damage to DNA [13,14,15]. Consequently, the measurement of MNi in lymphocytes has become one of the most widely applied genotoxicity testing methods for use with human cells both for in vitro genotoxicity testing and for human biomonitoring of exposure to genotoxins [16,17,18,19,20,21,22].

**Figure 1:**
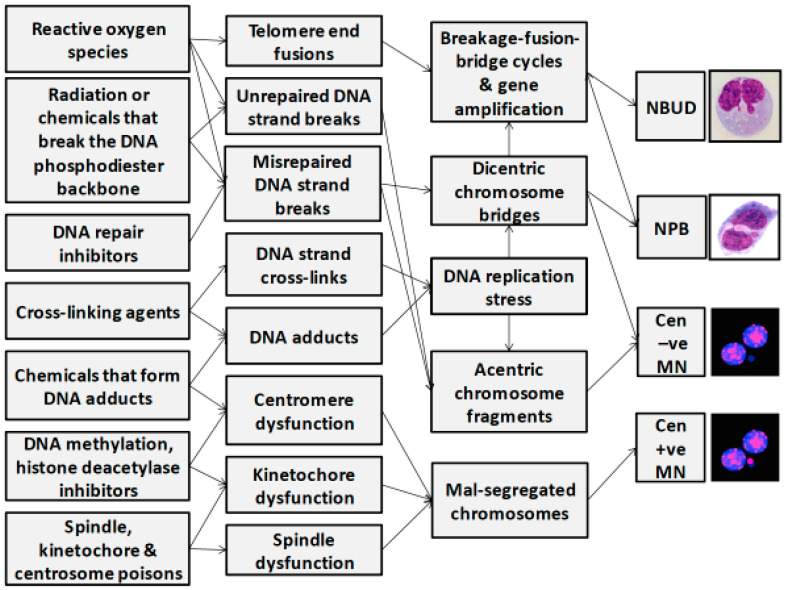
Molecular mechanisms that lead to the formation of micronuclei (MNi), nucleoplasmic bridges (NPBs), and nuclear buds (NBUDs) in lymphocytes. Cen +ve, centromere positive; Cen −ve, centromere negative.

Several recent reviews have been written on the detailed mechanisms by which damage to DNA and the mitotic machinery can cause micronucleus (MN) formation and generate genome chaos [15,20,23,24,25,26]. There are essentially six main causes for increase in MN formation: (i) genetic defects in the proteins required for mitosis and its checkpoints, (ii) genetic defects in DNA repair enzymes, (iii) excessive exposure to chemical genotoxins, (iv) excessive exposure to ionising radiation, (v) excessive endogenous genotoxins generated by stressed metabolic processes, and (vi) deficiency in the micronutrients required as cofactors for DNA replication and repair. Combinations of these causes of MN formation are likely to occur simultaneously, resulting in either additive or synergistic effects. Figure 1 provides examples of the multiple mechanisms by which genotoxins may induce lesions in DNA and the mitotic apparatus that lead to formation of MNi and other nuclear anomalies.

## 3. The Cytokinesis-Block Micronucleus Assay

**Figure 2:**
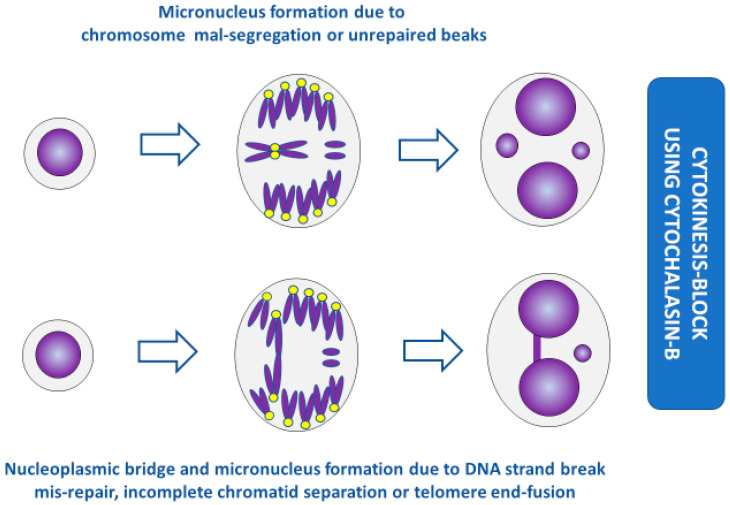
Formation of micronuclei and nucleoplasmic bridges in cytokinesis-blocked cells. (For the sake of simplicity, the conceptual diagrams only show a representative fraction of chromosomes.) Yellow dots represent centromeres. The acentric fragments in the bottom panel are generated during mis-repair of DNA strand breaks but not during incomplete chromatid separation or telomere end-fusion.

The expression of chromosome abnormalities as MNi requires completion of nuclear division, but prototype in vivo and in vitro MN assays do not distinguish between non-divided and divided cells. As a consequence, the results obtained by such methods are not reliable because the proportion of cells that complete nuclear division, and are therefore capable of expressing MNi, varies depending on culture conditions, biological age of the cells, and cytostatic or proliferative effects of the cytotoxic or genotoxic agent investigated that could delay mitotic progression or induce apoptotic cell death [27,28]. There was therefore a need to further develop the MN assay so that MNi could be scored specifically in cells that completed nuclear division. Different methods to achieve this were attempted but the one that was most reliable and adopted worldwide was the cytokinesis-block technique originally reported by Fenech and Morley [29]. In the cytokinesis-block micronucleus (CBMN) assay, cells that have completed nuclear division are accumulated and identified by their binucleated (BN) appearance after inhibiting the formation of the actin microfilament ring required for cytokinesis (Figure 2). Inhibition of cytokinesis and accumulation of BN cells is generally achieved by treating cell cultures with 3.0–6.0 μg/mL cytochalasin-B (Cyt-B) for 24–30 h. The concentration of Cyt-B and the duration of its treatment should be optimised for each cell type so that the frequency of BN cells is maximised. The latter is important for efficient scoring of MNi in BN cells.

## 4. The CBMN Cytome Assay to Measure Chromosomal Instability

**Figure 3:**
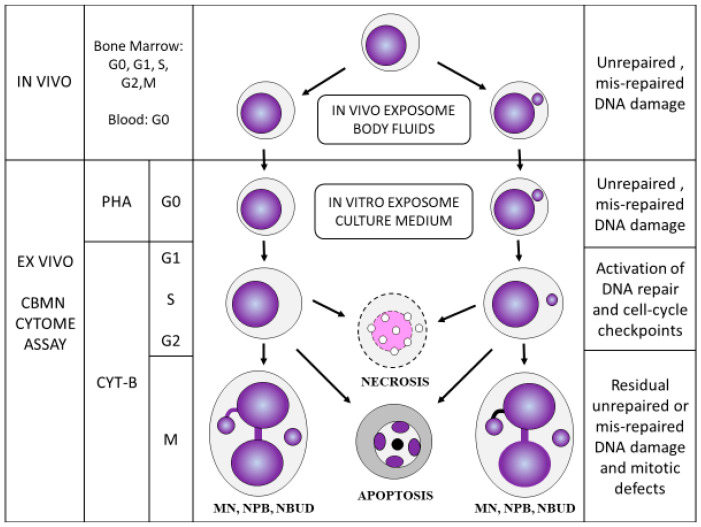
The main features of the lymphocyte cytokinesis-block micronucleus cytome (CBMNcyt) assay. G0, G1, S, G2, and M represent the various stages of the nuclear division cycle following stimulation using the mitogen phytohaemagglutinin (PHA). CYT-B, cytochalasin-B, which is used to block cells that complete nuclear division at the binucleated stage. MN, micronucleus; NPB, nucleoplasmic bridge; NBUD, nuclear bud. This figure is a copy of Figure 3 in Kirsch-Volders et al. [37]. Permission to re-use this figure was obtained from the publisher Elsevier.

**Figure 4:**
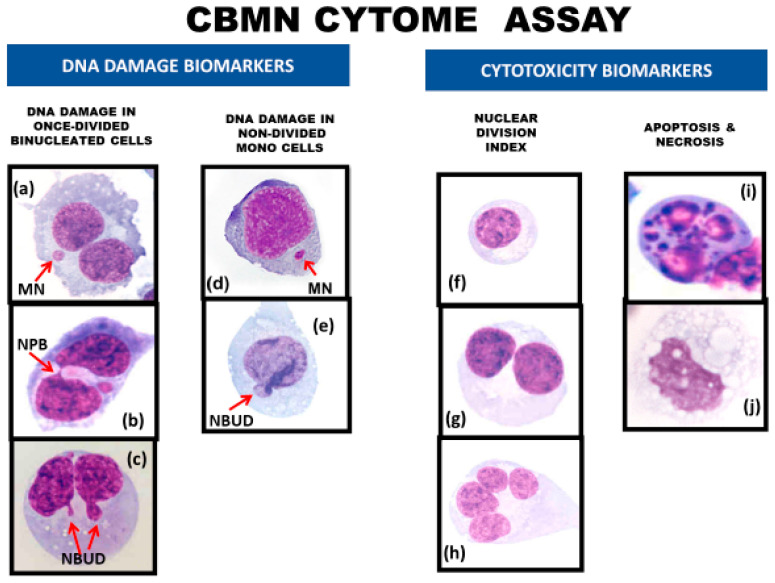
DNA damage and cytotoxicity biomarkers in the lymphocyte cytokinesis-block micronucleus cytome (CBMN-Cyt) assay. DNA damage biomarkers include (**a**) binucleated (BN) cells with a micronucleus (MN), (**b**) BN cells with a nucleoplasmic bridge (NPB), (**c**) BN cells with a nuclear bud (NBUD), (**d**) mononucleated (MONO) cells with a MN, and (**e**) MONO cells with a NBUD. Cytotoxicity biomarkers include the relative frequencies of (**f**) MONO cells, (**g**) BN cells, and (**h**) multi-nucleated cells with three or more nuclei, which together are used to measure the nuclear division index, a measure of cytostatic effects. In addition, (**i**) apoptotic cells and (**j**) necrotic cells are scored to obtain a measure of total cell death and the type of cell death. This figure has been adapted from Figure 1 in Rodrigues et al. [38]. Permission was obtained from the publisher Elsevier.

**Figure 5:**
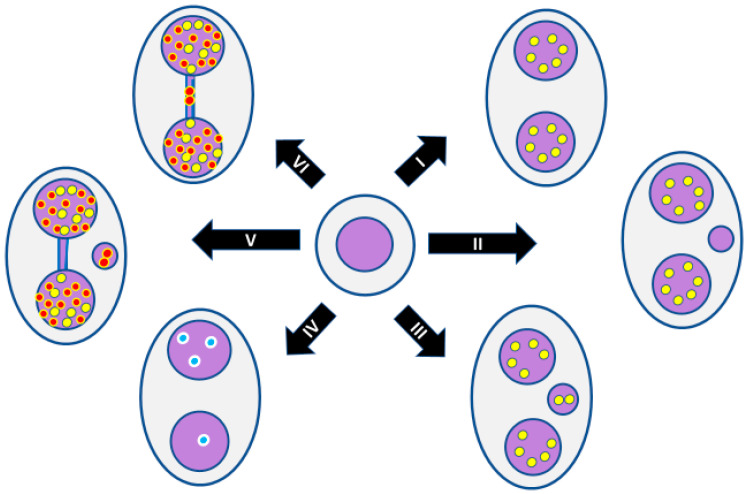
Use of molecular probes in the CBMN cytome assay to understand mechanisms: (**I**) normal binucleated (BN) cell; (**II**) BN cell containing one micronucleus originating from an acentric chromosome fragment; (**III**) BN cell containing one micronucleus originating from a mal-segregated whole chromosome; (**IV**) Mal-segregation of a chromosome in a BN cell without a micronucleus, which is evident because of unequal distribution of chromosome-specific peptide nucleic acid (PNA) probe signals; (**V**) BN cell with a nucleoplasmic bridge and micronucleus originating from a dicentric chromosome and an acentric chromosome fragment as a result of mis-repair of DNA strand breaks; (**VI**) BN cell with a nucleoplasmic bridge only, originating from a dicentric chromosome as a result of fusion of the telomeres of two chromosomes. Yellow dots represent signals observed using pancentromeric PNA probes. Red dots represent signals observed using telomere PNA probes. Blue dots represent signals observed using a PNA probe specific to only one chromosome. For the sake of simplicity, the conceptual diagrams only show a representative fraction of chromosomes.

**Figure 6:**
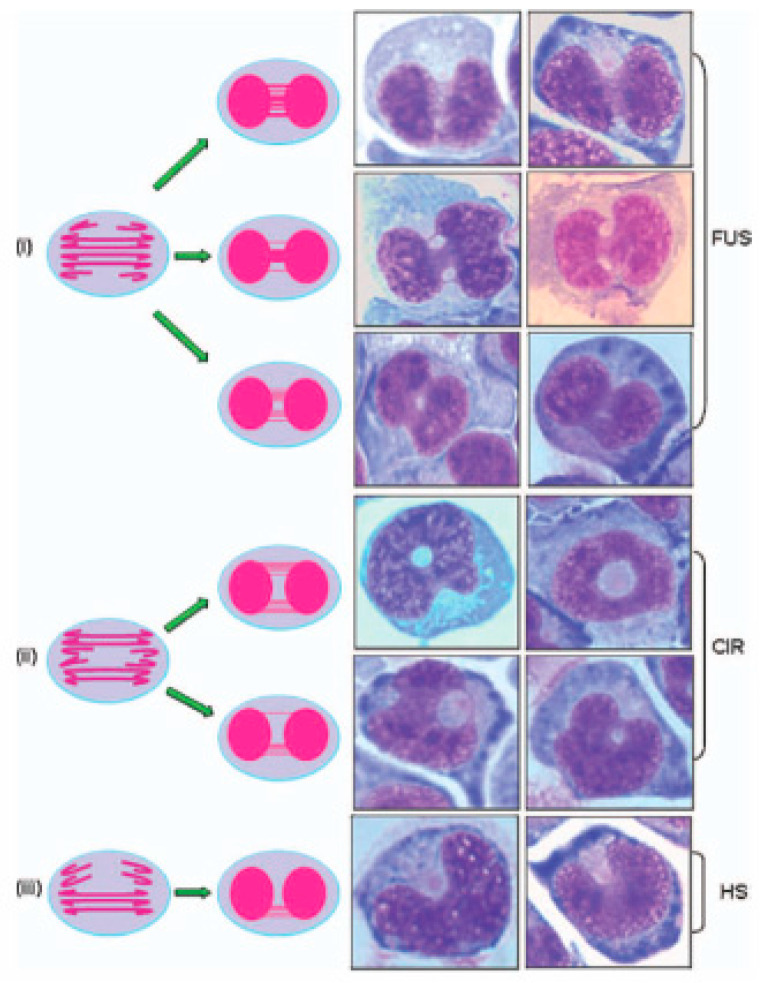
Typical appearances of the additional nuclear anomalies observed in the CBMN-Cyt assay. The nuclear anomalies: (**i**) fused (FUS), (**ii**) circular (CIR), or (**iii**) horseshoe (HS) nuclei. In the proposed models: (**i**) FUS nuclei originate as a result of multiple nuclear strands occurring uniformly or centrally between the nuclei of a binucleated (BN) cell, (**ii**) CIR nuclei originate as a result of multiple nuclear strands occurring on opposite sides between the nuclei of a BN cell, and (**iii**) HS nuclei originate as a result of multiple nuclear strands occurring only on one side between the nuclei of a BN cell. In each of these models, the total combined width of the connections between the nuclei in a BN cell is typically larger than one-fourth of the nuclear diameter—the maximum width of a conventional NPB, as defined within the CBMN-Cyt assay [13]. For the sake of simplicity, the conceptual diagrams only show a representative fraction of chromosomes. This figure was reproduced from Bull et al. [31], with permission from Wiley Periodicals, Inc.

Shortly after the invention of the CBMN assay, it became evident that, apart from the exclusive scoring of MNi in cells that completed nuclear division, there were other advantages to using the cytokinesis-block method:

1. Blocking of cells in the binucleated cell stage allowed the efficient measurement of the formation of nucleoplasmic bridges (NPBs) that originate from dicentric chromosomes caused by telomere end-fusions or mis-repair of DNA breaks or failure of complete chromatid separation (Figure 2, Figure 3, Figure 4, Figure 5 and Figure 6) [13,20,23,30,31].

2. Nuclei in BN cells form nuclear buds (NBUDs), which are another indicator of chromosomal instability as they are a mechanism through which nuclei eliminate amplified genes and unresolved DNA repair complexes (Figure 3 and Figure 4) [32,33,34].

3. The ability to score MNi, NPBs, and NBUDs within the same cells made it possible to reliably test the relationship between these biomarkers of chromosomal instability and discover that they are highly correlated with each other and represent the cytogenetic phenotype of bridge–breakage–fusion cycles that are initiated by the formation of dicentric chromosomes and cause gene amplification (Figure 2, Figure 3, Figure 4 and Figure 5) [30,31,35,36].

4. The ratios of mononucleated (MONO) cells, BN cells, and multi-nucleated (MULTI) cells with more than two nuclei (Figure 4) provided an index of cell proliferation and cytostasis [13].

In addition, it became evident that necrotic and apoptotic cells (Figure 4) could be easily identified using a simple set of scoring criteria so that the frequency and mechanism of cell death could also be efficiently measured and considered as an important factor in the toxicological assessment of the tested conditions.

## 5. Use of Molecular Probes in the CBMN Cytome Assay to Understand the Mechanisms

Molecular probes can further interrogate cells in the CBMN cytome assay to elucidate the mechanism of the formation of MNi, NPBs and NBUDs [39,40,41,42,43]. For example, pancentromere peptide nucleic acid (PNA) probes are now often used to determine whether MNi originated from an acentric chromosome fragment or a whole chromosome loss event, which would be centromere-negative and centromere-positive, respectively (Figure 5II,III) [39,40]. Furthermore, by using chromosome-specific PNA probes, it is possible to test, even in BN cells without a MN, whether non-disjunction of a specific chromosome has occurred by, for example, a distribution of the probe signal of 3:1 instead of 2:2 among the daughter nuclei (Figure 5IV) [41].

Telomere probes can also be used together with centromere probes. A MN that is telomere-positive but centromere-negative contains an acentric chromosome fragment and its associated telomere; in such an event, one of the daughter nuclei from the same mitosis will consequently have a defective chromosome that lacks the fragment and telomere trapped in the MN (Figure 5V). An NPB that is telomere-positive in the bridge region between centromeres most likely originates from telomere end-fusion (Figure 5VI), whereas an NPB that is telomere-negative in the bridge region (Figure 5V) is probably the result of mis-repair of sub-telomeric DNA breaks involving two chromosomes, which generates a dicentric chromosome and an acentric chromosome fragment that is telomere-positive; the latter results in formation of a telomere-positive MN. Using telomere and centromere probes, it is possible to determine the origin of MNi and NBUDs induced by folate deficiency [42], with MNi being primarily derived from lagging chromosomes (22.0%) and terminal acentric fragments (62.2%), whereas most NBUDs originate mainly from interstitial fragments (42.7%) or terminal acentric fragments (43.5%).

## 6. Other Emerging Biomarkers in the CBMN Cytome Assay

Other nuclear anomalies can be observed in cells with chromosomal instability, but these have not yet been adequately validated. For example, we have identified additional nuclear anomalies formed under folate-deficient conditions, defined as fused (FUS), circular (CIR), and horseshoe (HS) nuclei, and investigated their suitability for inclusion as additional CIN biomarkers in the lymphocyte cytokinesis-block micronucleus cytome (CBMN-Cyt) assay (Figure 6) [31,44]. Although the morphological appearance of FUS, CIR, and HS nuclei suggested an origin from multiple NPBs in the fusion region between the two nuclei, the low frequency of dicentric chromosomes in the metaphase spreading from these cultures did not support this model [31]. Fluorescence in situ hybridization (FISH) analysis of cytokinesis-blocked binucleated (BN) cells with peptide nucleic acid probes for telomeres and centromeres (PNA-FISH) revealed a high proportion of fusion regions containing both centromeric and telomeric DNA. This suggested that folate deficiency may disrupt the process of sister chromatid separation and chromosome segregation during mitosis. It was concluded that the FUS, CIR, and HS morphologies represent promising biomarkers of CIN that are sensitive to folate deficiency. Further validation and investigation of the mechanisms responsible for their formation is warranted.

Reports have recently emerged indicating that mutations in genes that affect cohesin, condensin, and separase functions, which control chromatid separation during mitosis, cause multiple anaphase bridges similar to those that may result in formation of FUS, CIR, and HS nuclei [45,46,47,48]. Furthermore, a proportion of the multiple narrow bridges observed between the nuclei of FUS cells may also be ultra-fine bridges (UFBs), which can arise from DNA catenanes at centromeres/rDNA loci, late replication intermediates induced by replication stress, and DNA linkages at telomeres [49]. However, this has not been previously tested and would require the detection of proteins that specifically bind UFBs, including Bloom’s syndrome helicase (BLM), PLK1-interacting checkpoint helicase (PICH) and replication protein A (RPA), to verify their involvement [49].

## 7. Shattering of Chromosomes in MNi and Their Subsequent Hypermutation and Inflammation

Another important recent development regarding the consequences of MN formation is the observation that entrapment of chromosomes in MNi may result in premature chromosome condensation of the chromosome and its fragmentation. This situation occurs when DNA replication in the MN is delayed relative to the nucleus [50,51,52,53,54,55,56]. The resulting chromosome fragments may be incorporated into one of the daughter nuclei and undergo error-prone repair by non-homologous end joining, resulting in a massively rearranged mutant chromosome [52,53,57,58,59,60]. Therefore, it is evident that MNi are not only biomarkers of induced chromosomal instability but also a cause of further amplification of chromosomal instability that can occur in just two cell cycles.

One of the reasons for delayed DNA replication in MNi could be because of DNA replication stress resulting from lack of the enzymes and cofactors required for DNA synthesis and repair. This may be due to defective nuclear envelope assembly of MNi resulting in a lack of nuclear pore complexes; consequently, MNi fail to properly import key proteins that are necessary for the integrity of the nuclear envelope and the genome [54,61,62,63]. Disruption of the MN membrane may also either result in leakage of DNA into the cytoplasm and/or provide access for cytoplasmic DNAases to attack the DNA in MNi [54,62,63]. There is now a substantial body of evidence that DNA from MNi can be sensed by the innate immune system via cyclic GMP-AMP synthase (cGAS), which generates Cyclic guanosine monophosphate–adenosine monophosphate (cGAMP), which in turn activates Stimulator of Interferon Genes (STING), resulting in increased expression of interferon and activation of pro-inflammatory interferon-stimulated genes [64,65,66,67]. The induced inflammation, if left unresolved, can subsequently increase oxidative stress and further aggravate chromosomal instability, resulting in a vicious cycle of MNi, NPBs, NBUD formation, hypermutation, and increasing inflammation.

Methods to measure the pulverisation of chromosomes trapped in MNi, defective membranes in disrupted MNi, cGAS positive MNi, and leakage of DNA into the cytoplasm in cells containing MNi have been recently published [50,51,52,53,54,55,56,57,58,59,60,61,62,63,64,65,66,67].

**Figure 7:**
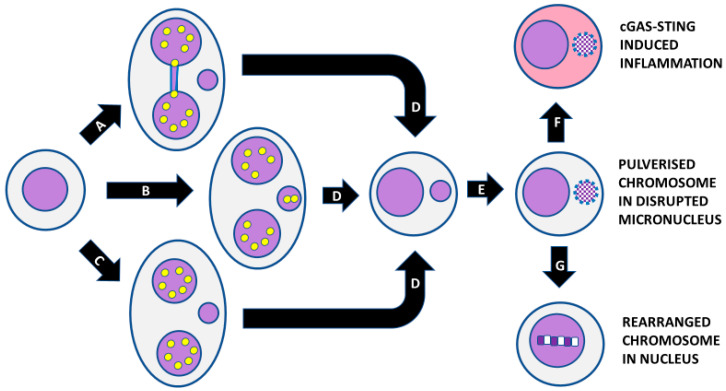
Consequences of micronucleus formation. Micronucleus formation at the binucleated cell stage as a result of (**A**) mis-repair of DNA breaks leading to a nucleoplasmic bridge and an acentric chromosome fragment, (**B**) the whole chromosome as a result of mal-segregation, and (**C**) lagging acentric chromosome fragments as a result of unrepaired DNA breaks. (**D**) Mononuclear cell with a micronucleus after completion of cytokinesis. (**E**) Shattering of chromosomes trapped in a micronucleus and disruption of the nuclear envelope of the micronucleus. (**F**) Leakage of DNA from the micronucleus, leading to activation of cyclic GMP-AMP synthase–Stimulator of Interferon Genes (cGAS-STING) mechanism and induction of inflammatory cytokines. (**G**) Integration of pulverised chromosomes within the main nucleus and error-prone repair by non-homologous end-joining, resulting in a greatly rearranged mutant chromosome. Yellow dots represent centromeres.

Figure 7 summarises the key steps in MNi formation, the pulverisation of chromosomes entrapped in MNi, and the consequent steps of either hypermutation of the trapped chromosome and/or the triggering of inflammation by the cGAS-STING mechanism.

## 8. Conclusions and Future Directions

The CBMN cytome assay is a multi-endpoint cytogenetic technique that enables measurement of several nuclear anomalies indicative of many aspects of CIN, which include structural/numerical chromosome aberrations and chromosome mal-segregation during mitosis expressed as MNi, anaphase bridge formation expressed as NPBs, and gene amplification or elimination of unresolved DNA complexes expressed as NBUDs.

Mechanistic information about the origin of MNi, NPBs. and NBUDs can be obtained by the additional use of molecular probes such as those that detect the presence of centromeres, telomeres, and DNA damage response proteins.

The consequences of chromosome entrapment within MNi are chromothripsis caused by premature chromosome condensation in later replicating MNi and chromoanagenesis due to error-prone repair of the resulting shattered chromosomes.

The additional consequence of MNi formation is stimulation of the innate immune system’s cGAS-STING pathway, which further increases the importance of MNi formation as a biomarker of cellular malfunction and as a driver of degenerative diseases that increase with age.

In view of the above, it is important that future research addresses the following questions:

(1) What are the preventable causes of MNi, NPBs, and NBUD formation?
(2) What are the most important genetic causes of MNi, NPBs, and NBUD formation?
(3) Are cGAS-STING induction and chromoanagenesis mutually exclusive or do they occur one after the other in the same cell?
(4) What are the molecular differences between MNi that remain extranuclear and trigger cGAS-STING versus MNi that result in chromothripsis followed by nuclear reintegration and chromoanagenesis?
(5) What are the best probes to detect chromosome pulverisation in MNi, induction of cGAS-STING by MNi, and chromoanagenesis of a pulverised chromosome within a nucleus?
(6) Is it possible to further enhance the lymphocyte CBMN cytome assay by automation so that it becomes practical to use it as a high-content molecular cytogenetic assay using multiple probes simultaneously not only as a research tool to study mechanisms but also in routine clinical diagnostics of healthy aging?

## Abbreviations

BN	binucleated
CBMN	cytokinesis-block micronucleus
cGAS	cyclic GMP-AMP synthase
CIN	chromosomal instability
FISH	fluorescence in situ hybridisation
MN	micronucleus
MNi	micronuclei
NBUDs	nuclear buds
NPBs	nucleoplasmic bridges
PNA	peptide nucleic acid
RBCs	red blood cells
STING	Stimulator of Interferon Genes
UFBs	ultra-fine bridges
